# Estimation of milk yield based on udder measures of Pelibuey sheep using artificial neural networks

**DOI:** 10.1038/s41598-022-12868-0

**Published:** 2022-05-30

**Authors:** J. C. Angeles-Hernandez, F. A. Castro-Espinoza, A. Peláez-Acero, J. A. Salinas-Martinez, A. J. Chay-Canul, E. Vargas-Bello-Pérez

**Affiliations:** 1 Instituto de Ciencias Agropecuarias, Universidad Autónoma del Estado de Hidalgo, 43600 Tulancingo, Hidalgo Mexico; 2 Instituto de Ciencias Básicas e Ingeniería, Universidad Autónoma del Estado de Hidalgo, 42184 Pachuca, Hidalgo Mexico; 3 División Académica de Ciencias Agropecuarias, Universidad Juarez Autonoma de Tabasco, 86290 Villahermosa, Tabasco Mexico; 4 Department of Veterinary and Animal Sciences, Faculty of Health and Medical Sciences, University of Copenhagen, Grønnegårdsvej 3, 1870 Frederiksberg C, Denmark

**Keywords:** Animal physiology, Biomechanics

## Abstract

Udder measures have been used to assess milk yield of sheep through classical methods of estimation. Artificial neural networks (ANN) can deal with complex non-linear relationships between input and output variables. In the current study, ANN were applied to udder measures from Pelibuey ewes to estimate their milk yield and this was compared with linear regression. A total of 357 milk yield records with its corresponding udder measures were used. A supervised learning was used to train and teach the network using a two-layer ANN with seven hidden structures. The globally convergent algorithm based on the resilient backpropagation was used to calculate ANN. Goodness of fit was evaluated using the mean square prediction error (MSPE), root MSPE (RMSPE), correlation coefficient (r), Bayesian’s Information Criterion (BIC), Akaike’s Information Criterion (AIC) and accuracy. The 15–15 ANN architecture showed that the best predictive milk yield performance achieved an accuracy of 97.9% and the highest values of r^2^ (0.93), and the lowest values of MSPE (0.0023), RMSPE (0.04), AIC (− 2088.81) and BIC (− 2069.56). The study revealed that ANN is a powerful tool to estimate milk yield when udder measures are used as input variables and showed better goodness of fit in comparison with classical regression methods.

## Introduction

Traditionally sheep rearing in tropical regions of Latin America is focused on lamb production. However, recently, farmers have increased their interest on diversifying sheep products. Pelibuey is a breed used mainly for meat production as it has maternal ability and prolificity, and over the last decade its milk production performance has been explored^1^. Milk production and milk composition from Pelibuey sheep has been analyzed as pure breed^2^ and as a crossbred with specialized dairy breeds^1^.The improvement of milk production in this breed requires milk yield recording and in order to favor an adequate productive performance, determination of udder morphological characteristics is needed^2^.

In sheep as in other dairy animals, the anatomical characteristics of udder are important for milk production and milkability^3^. The mammary gland morphology is related with milk production and aptitude for mechanical milking, which has already been used in genetic programs for dairy sheep^4,5^. For instance, Rovai et al.^6^ suggested that sheep with greater milk yields showed large cisternal udders that were able to secrete high milk volumes. In the same line, McKusick et al.^7^ reported that increasing one centimeter of udder circumference and udder height results in an increased from 0.06 to 0.11 L of milk in East Friesian ewes. This relationship allows to estimate milk yields from the udder measures.

Several approaches have been used to estimate milk production based on morphological measures of the udder and those have yielded to large differences in their predictive accuracy. The ability to estimate dairy performance depends on the number and type of udder measures, stage of lactation and analytical tools used to assess the udder-milk yield relationship^8^. Analyzing the relationships between udder morphology and milk yield from dairy ewes started back in the 1950s, and until now, all studies focused on this topic have analyzed this relationship using classical estimation methods^9,10^, without exploring new computational analyzing tools that increase the ability to explain milk yield performance based on udder measures. In this regard, most of the studies use multiple regressions as an approach to estimate milk production using udder measures as main predictor. However, these traditional approaches have several limitations such as sensibility for missing data, need of normal distribution data, and overparameterization when many predictors are used, which limits their use and fitting performance.

The used artificial neural networks (ANN) are capable to deal with complex non-linear relationships between input and output variables, with no need for rigid a priori models^11^. The AAN is a nonlinear information processing system inspired by the biological nervous system. The ANN is based on interconnected elementary processing devices called neurons. The networking begins from the input information through one (or) more hidden layers, to the input layers. The ANN application has several advantages as good self-learning ability, nonlinearity, massive parallelism, easy implementation, adaptability, real-time operation, and fault tolerance ability^12,13^. The ANN has been used in animal science for classification and prediction purposes related to nutritional management^13,14^, dairy performance^15,16^, animal health^17^, genetic improvement^11,18^ and processing of dairy products^19^. However, in ruminants there is no evidence of their used to estimate the relationship between milk yield and mammary gland measures in Pelibuey ewes.

The ANN are flexible models in terms of model assumptions and structure with accurate and precise predictions^13^. However, its use is challenged by defining optimal neuron network architecture, which implies that the determination of hidden units required to mimic the analyzed system using input–output examples is needed. The approach to this issue requires considering the relation between the model complexity and training performance. For example, using Akaikes’s Information Criterion (AIC) and other criteria of goodness of fit allow choosing adequate ANN architecture^20^. Therefore, the aim of this study is to assess the different ANN architectures applied to udder measures from Pelibuey ewes to estimate milk yield and comparing them with predictions from linear regressions.

## Results

**Figure 1:**
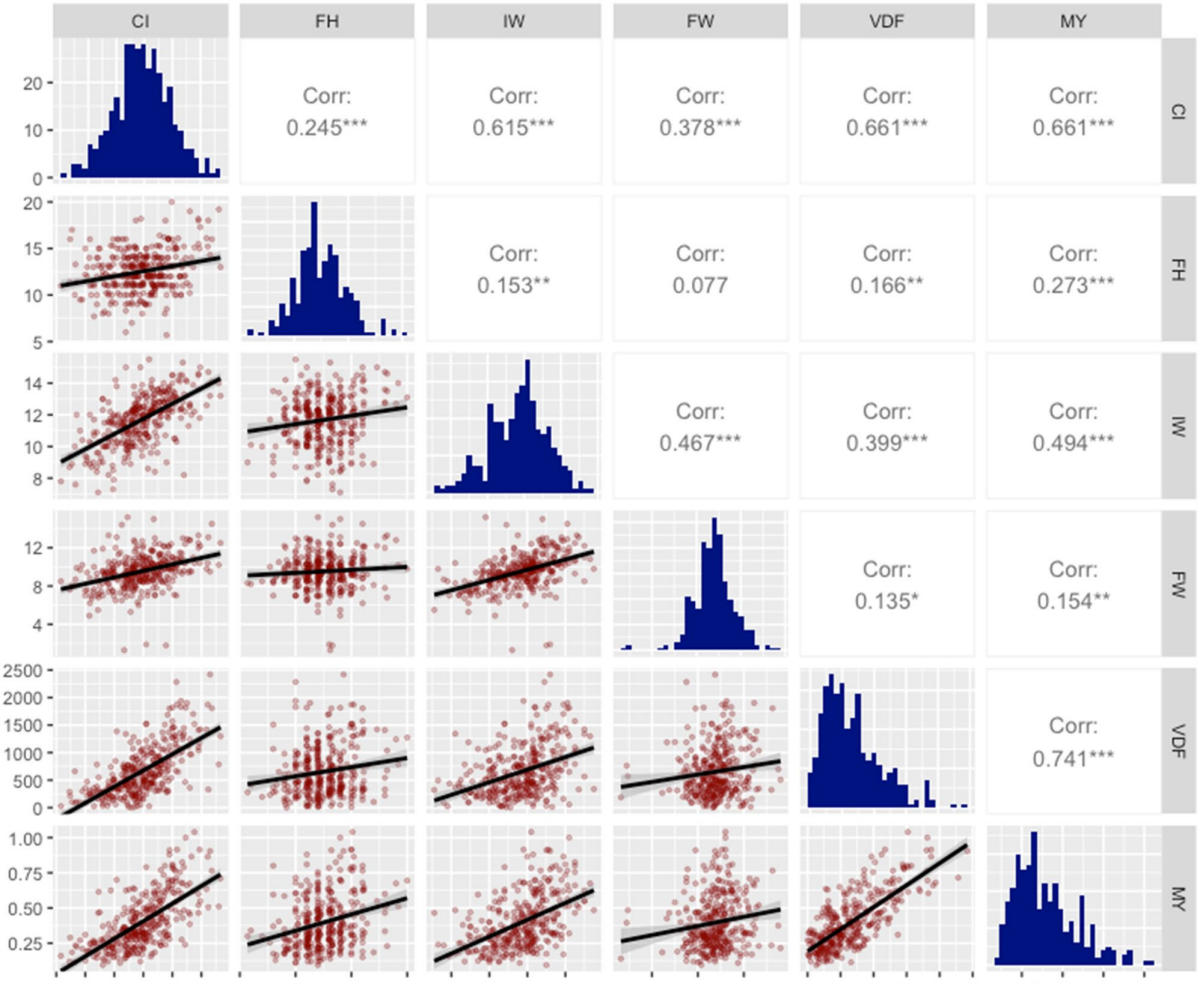
Analysis of the input and output variables. Scatterplots, distributions, and correlation coefficients of initial udder circumference (CI), final udder heigh (FH), initial udder width (IW), final udder width (FW), difference initial and final udder volume (VDF) and milk yield (MY).

Scatterplots, distributions and correlation coefficients of input and output variables are shown in Fig. 1. Must variables had a normal distribution, but the VDF and MY showed right skewness. The scatterplots showed significant linear relationship between the inputs CI (r = 0.66; *p* < 0.001) and VDF (r = 0.741; *p* < 0.001) with the output MY. Also, a significant correlation between CI and IW (r = 0.615; *p* < 0.001) and VDF (r = 0.661; *p* < 0.001) was found, which could determine the presence of multicollinearity in the MLR implementation.

**Figure 2:**
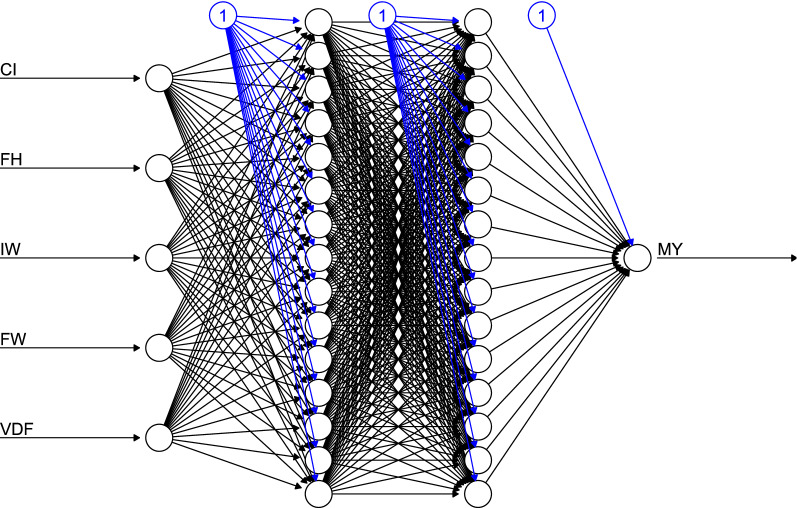
Architecture of used ANN with two-layers and 15 neurons to estimate milk yield (MY) and initial udder circumference (CI), final udder heigh (FH), initial udder width (IW), final udder width (FW), and difference initial and final udder volume (VDF) as input variables.

Table 1 shows the goodness of fit of MRL and the seven hidden configurations of the ANN. The proportion of the variance of MY explained by MRL was of 62.0%, with an accuracy of 90.6% and a value of RMSPE of 0.11 kg. The ANN with an architecture of two layers with five neurons (5–5) each one showed similar values of goodness of fit criteria and MLR accuracy. However, with the increase of neurons in both layers improved the goodness of fit. This finding indicated that modifying the number of hidden nodes influenced the model performance and its estimations. Figure 2 shows the best ANN architecture, which was structured by two-layers and 15 neurons each one (15–15). The 15–15 ANN architecture achieved an accuracy of 97.9% and depicted the highest values of r (0.97) and r^2^ (0.93), and the lowest values of RMSPE (0.04 kg), AIC (− 2088.81) and BIC (− 2069.56).

**Table 1:** Evaluation of goodness of fit of multiple linear models and artificial neural networks used to predict milk yield of Pelibuey sheep.

Model	r	r^2^	RMSPE	AIC	BIC	Accuracy
MLR	0.79	0.62	0.11	− 1495.96	− 1476.72	90.6
Hidden*						
5–5	0.80	0.64	0.11	− 1507.05	− 1487.81	90.5
5–10	0.86	0.79	0.09	− 1612.88	− 1593.63	92.8
10–5	0.89	0.73	0.09	− 1688.69	− 1669.44	94.3
10–10	0.86	0.74	0.09	− 1616.60	− 1597.35	93.6
10–15	0.95	0.89	0.06	− 1942.49	− 1923.24	97.3
15–10	0.91	0.83	0.07	− 1776.54	− 1757.30	95.4
15–15	0.97	0.93	0.04	− 2088.81	− 2069.56	97.9

*MRL* multiple linear regression model, *r* correlation between actual and predicted values, *r*^2^ Adjusted R-squared, *RMSPE* root mean square prediction error, *AIC* Akaike’s Information Criterion, *BIC* Bayesian’s Information Criterion.

*Number of neurons layer 1- number of neurons layers 2.

**Figure 3:**
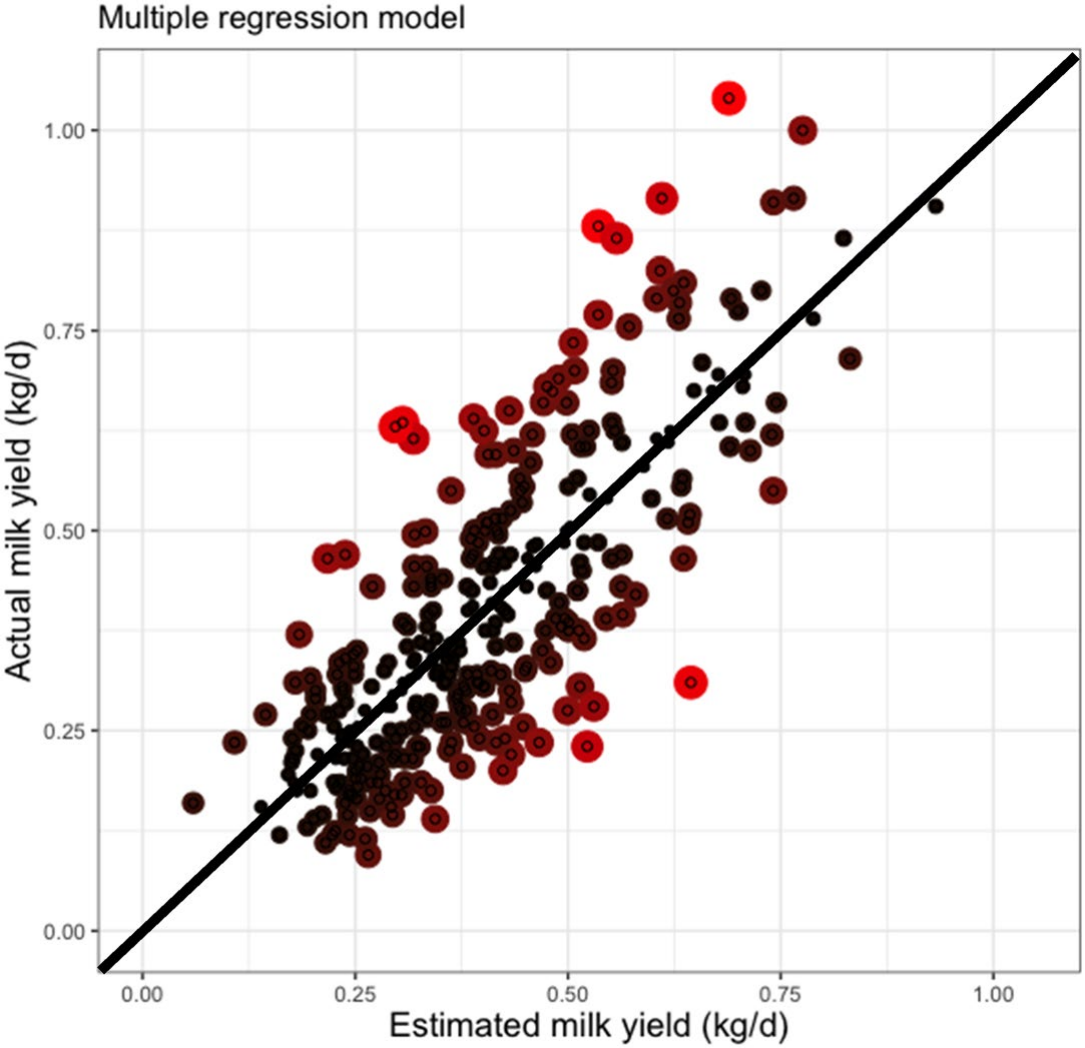
Residual analysis of multiple regression model to estimate milk yield using morphometric measures of udder from Pelibuey sheep. The color and size of circles represents the magnitude of residuals. Black and red color means a lower and higher residual values, respectively.

**Figure 4:**
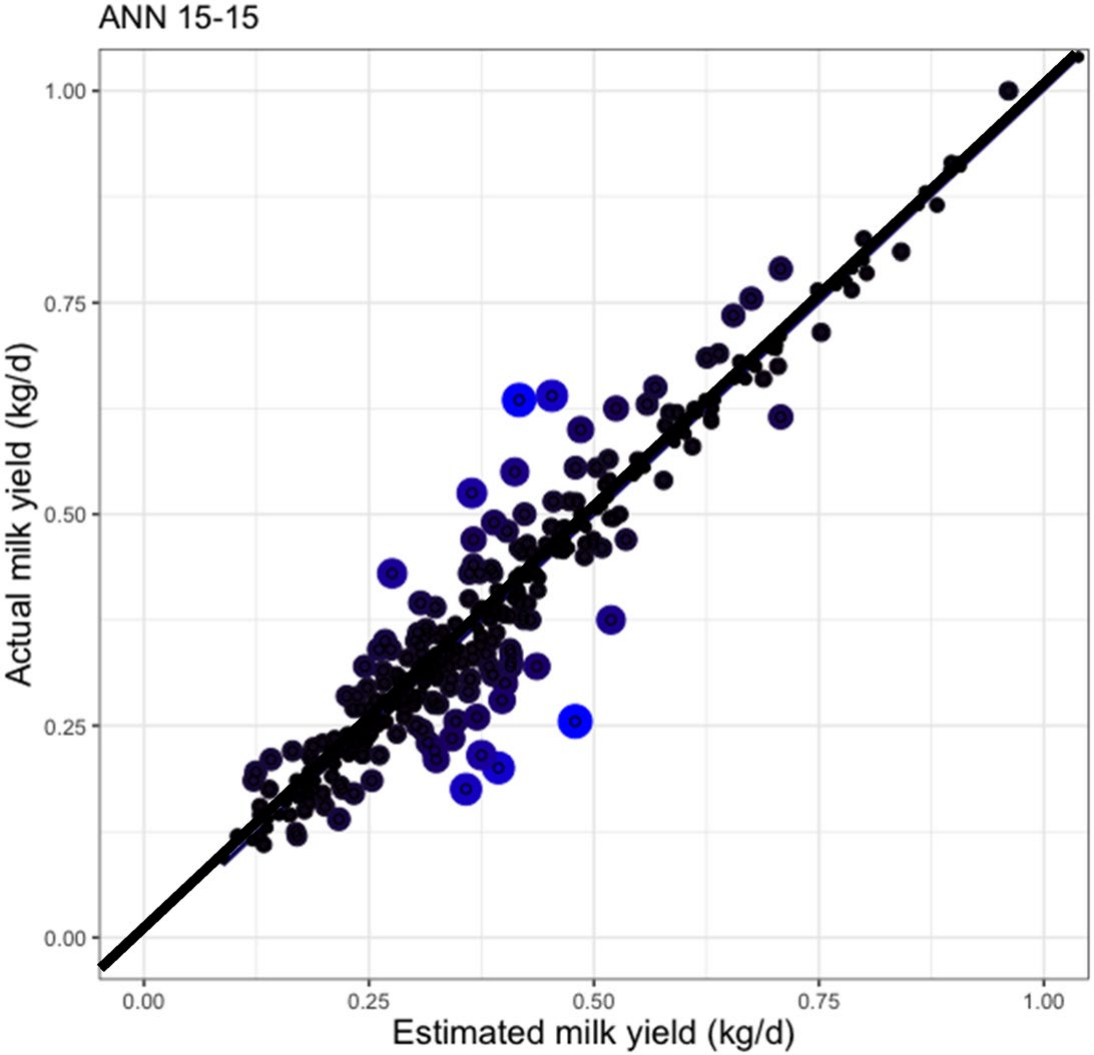
Residual analysis of ANN with two-layers (15–15 neurons) used to estimate milk yield using morphometrics measures of udder in Pelibuey sheep. The color and size of circles represents the magnitude of residuals. Black and blue color means a lower and higher residual values, respectively.

Figures 3 and 4 shows the residual plots of the MRL and 15–15 ANN models used to estimate milk yield. According to their size, and shown as red colored of circles, in Fig. 3, the MRL model showed the worst estimations of milk yields that were around 0.75 and 1.0 kg. In contrast, the ANN 15–15 showed a lower goodness of fit when estimated milk yields were around 0.25 and 0.5 kg. Finally, when the magnitude of residuals was compared with MRL and ANN 15–15, the artificial intelligence approaches showed lower values along with all milk yield estimations.

## Discussion

Our study assessed the usefulness of udder measures to estimate milk production on Pelibuey sheep using machine-learning techniques. The accurate prediction of milk yield is a fundamental step for implementing strategies to improve dairy performance in small ruminants. Using milk yield records, the ANN have been applied to estimate milk yield in dairy cows^15,21,22^, sheep^23^, goat^24,25^ and buffalo^26^. The udder measures have been widely used to predict milk yield^2^; however, these predictions have been carried out using traditional statistical tools such as multiple linear regression. The current study is the first reporting the use of ANN to estimate milk yield based on udder measures from sheep.

Several studies have compared the performance of traditional predictive tools (i.e., multiple linear regression and random forest) versus ANN to estimate milk yield^15,21^, predict mastitis^27^, forecasting cow locomotion score^28^, prediction of body weight^29,30^, modelling rumen fill^31^ and prediction of carcass tissue composition^32^. Compared with MLR, results from the current study revealed a better performance of ANN to estimate milk yield based on udder measures. These findings agree with Bhosale and Singh^21^ who reported that the performance of ANN (r^2^ = 83.5) was better than the MLR (r^2^ = 76.21) model for milk yield prediction. In addition, other authors have reported a better performance of ANN versus MLR when estimating milk yields from ruminants^15,33,34^.

In the current study, an increase of goodness of fit and accuracy was observed as number of nodes in the two hidden layers were increased. Which is explained by the fact that the number of nodes in the hidden layers defines the complexity and power of the neural network model to delineate underlaying relationships and structures inherent to the database. The number of nodes in the hidden layers should be large enough for the correct representation of the studied phenomena, but at same time low enough to have adequate generalizations. Several methods have been proposed to define the optimum number of hidden layer nodes. For instance, the approach proposed by Garson^35^ is often used to choose the number of hidden neurons and is calculated as follow: Np/[r(Ni + No)]; where ﻿Ni and No are ﻿input and output layer nodes, respectively, and the number of training samples is represented by Np. The Garson^35^ approach determines an optimal number of layers to our database in a range of 6 to 12 nodes, which is markedly lower than the best architecture tested in the current study (n = 30).

Recently, Madhiarasan and Deppa^12^ tested 151 different criteria of selection of the number of hidden neurons in back propagation networks using the RMSE, Mean Absolute Error (MAE), Mean Relative Error (MRE) and Mean Square Error (MSE) as error criteria to choose the optimal neuron network architecture. These authors also proposed a new approach when selecting the number of hidden neurons: = (8*n* − 4)/(*n* − 4) where *n* is the number of input parameters. The use of this new approach allows selecting the model of ANN with the lowest error value of forecasting and easy implementation. For the current database, the computed number of hidden neurons using the Madhiarasan and Deppa^12^ approach was 36, which is similar to the best architecture tested in the current study.

Therefore, we considered that an ANN architecture with 2 layers and 15 neurons in each one is the most appropriate for our database structure as it provides the best estimations without affecting its generalization capacity. Our best architecture had the highest number of hidden neurons, which is in accordance with Ince and Sofu^23^, who referred that a bigger network size would contribute to lower error levels in the training set. However, several authors referred that a more complex ANN architecture not necessarily represents an optimal solution for a given problem^15^. For this reason, the definition of the most appropriate architecture is the main step of the ANN implementation, which must considerer the context of research, database structure, type of variables, learning methods and objective of implementation (prediction or classification)^15,23^.

Overall, five udder measures were used to estimate the milk yield from Pelibuey sheep implementing two analytic approaches: multiple linear regression (MLR) and artificial neural networks (ANN). Both approaches were able to predict milk yield. However, the ANN showed better goodness compared with MLR. The accuracy of ANN to estimate milk yield depended on its neuron network architecture with increasing accuracy as the complexity of the ANN was increased. Therefore, the result of the current study reveals that ANN is a powerful tool to estimate milk yield when udder measures are used as an input variable and our data could be used or extrapolated for studies related to dairy ruminants. Further studies could use larger datasets and use them to perform different experiments including hyperparameter optimization by applying different approaches such as grid search, meta-heuristics, evolutionary methods and those based on nature-inspired algorithms.

## Methods

Animal care, welfare and procedures were carried out according to the guidelines of the Animal Care Committee of the División Académica de Ciencias Agropecuarias, Universidad Juárez Autónoma de Tabasco under the approved ID project PFI: UJAT-DACA-2015-IA-02.

### Database

A total of 357 milk yield records with its corresponding gland udder measures was used in the present study. The records of a total of 38 multiparous Pelibuey ewes were analyzed in the current study. Ewes had an average body weight of 36.3 ﻿ ± 4.9 kg with single (n = 33) and twin lambing (n = 5). Details about the nutritional, health and lamb management are specified by Arcos-Álvares et al.^2^. Ewes were milking manually once a day and udder measures were recorded twice a week. Udder measures were taken before and after milking and those were udder circumference (circumference at the udder medium area), udder width (distance between the widest lateral points of the udder), udder height (distance from the udder insertion to the lower extremity) and udder volume were calculated as follows:

1 $$R = \frac{CP}{{2{\uppi }}}$$

2 $$V = \pi R^{2} Ud$$

where V is the udder volume (cm^3^), R is radius (cm), CP is circumference perimeter (cm), $\pi$ is 3.14159265358979 and Ud is the udder depth (cm).

### Model training

Arcos-Álvares et al.^2^ using the same database of the current study to estimate milk yield through fit several multiple linear regression (MLR) models. To estimate daily milk yield (MY), these authors used a stepwise process to choose the best model using the following goodness of fit criteria: mean square prediction error (MSPE), root of MSPE (RMSPE) and coefficient of determination (r^2^). For that, the inputs were initial udder circumference (CI), final udder heigh (FH), initial udder width (IW), final udder width (FW), and the difference between the initial and final udder volumes (VDF) as input variables. Arcos-Álvares et al.^2^ used five models with the best goodness of fit following this equation:

3 $$\begin{aligned} {\text{MY}} &= \, - 0.{34 }\left( { \pm \, 0.{66}^{***}} \right) \, + \, 0.00{7 }\left( { \pm \, 0.00{1}^{***}} \right) \, \times {\text{ CI }} + \, 0.00{9 }\left( { \pm 0.00{2}^{*}} \right) \times {\text{ FH}} \\ &\quad + \, 0.0{2 }\left( { \pm \, 0.00{5}^{***}} \right) \times {\text{ IW }} - \, 0.00{9 }\left( { \pm \, 0.00{4}^{*}} \right) \, \times {\text{ FW }} + \, 0.000{2 }\left( { \pm \, 0.0000{1}^{***}} \right) \, \times {\text{ VDF}} \\ \end{aligned}$$

where MY is the daily milk yield and the input variables (CI, FH, IW, FW and VDF) as were described above. The asterisks mean the level of significance of the regression coefficients: ﻿*p < 0.05; ***p < 0.000.

In order to contrast MLR versus ANN the same inputs were used to carried out machine learning essay. Normalization is very important to build ANN since the database has different range and different units. The proposed approaches use the min–max normalization technique using the formula proposed by Zeng and Yeung^36^:

4 $$X_{i}^{\prime } = \left( { \frac{{X_{i} - X_{min} }}{{X_{max} }} - X_{min} } \right)\left( {X_{max}^{\prime } - X_{min}^{\prime } } \right) + X_{min}^{\prime }$$

where *X*_*i*_ is the real input data, *X*^′^_*min*_ is the least input data, *X*^′^_*max*_ is the greatest input data, *X*^′^_*min*_ is the least target value, *X*^′^_*max*_ is the greatest target values.

A supervised learning was used to train and teach the network using a two-layer ANN with seven hidden structures: 5–5, 5–10, 10–5, 10–10, 10–15, 15–10 and 15–15 neurons. Inputs vales are transferers to the hidden layer that multiplies weight W using tangent sigmoid activation function; the outputs of hidden layers get transferred to the output layers that multiplies with weight V using tangent sigmoid activation function^12^. A random 70% of the data was used to training and 30% for testing, the process was repeated 100 times. The globally convergent algorithm based on the resilient backpropagation was used to calculate ANN with a maximum step for training ANN of 1 × 10^7^ using the *neuralnet* package in R software^37^.

### Performance assessment

The fit of ANN was compared with the best multiple regression model (MRM) fitted previously by Arcos-Alvarez et al.^2^ using same input variables in both models. Goodness of fit was evaluated using:

Mean square of prediction error (MSPE), calculated as:

5 $${\text{MSPE}} = \user2{ }\frac{{\mathop \sum \nolimits_{{{\varvec{t}} = 1}}^{{\varvec{n}}} {\varvec{e}}_{{\varvec{t}}}^{2} }}{{{\varvec{n}} - {\varvec{p}}}}$$

where *e*_t_ is the residual for the milk yield of *t* sheep, *n* is the number of observations in the training dataset and *p* is the number of parameters of models.

Root-mean-square prediction error (RMSPE):

6 $${\text{RMSPE}} = \sqrt {MSPE}$$

Akaike’s Information Criterion (AIC) calculated as follows^38^:

7 $${\text{AIC }} = {\text{ n }} \times {\text{ LL }} + { 2 } \times {\text{ k}}$$

where n is the number of examples in the training dataset, LL is the log-likelihood for the model using the natural logarithm (e.g., the log of the MSPE), and k is the number of parameters in the model.

Bayesian’s Information Criterion (BIC) calculated as follows^39^:

8 $${\text{BIC }} = {\text{ n }} \times {\text{ LL }} + {\text{ k }} \times {\text{ log}}\left( {\text{n}} \right)$$

where n is the number of observations in the training dataset, LL is the log-likelihood for the model using the natural logarithm (e.g. log of the mean squared error), and k is the number of parameters in the model, and log() is the natural logarithm.

Accuracy of estimations was calculated as follow:

9 $${\text{Accuracy}} = \, \left( {{\text{actual value}}-{\text{estimated value}}} \right)/{\text{actual value }} \times { 1}00$$

### Ethics declaration

Animal care, welfare and procedures were carried out according to the guidelines of the Animal Care Committee of the División Académica de Ciencias Agropecuarias, Universidad Juárez Autónoma de Tabasco under the approved ID project PFI: UJAT-DACA-2015-IA-02.

## Supplementary Information

Supplementary Information.

## Data Availability

Data is available as supplementary material.

## References

[1] Angeles-Hernandez JC, Ortega OAC, Perez AHR, Ronquillo MG (2014). Effects of crossbreeding on milk production and composition in dairy sheep under organic management. Anim. Prod. Sci..

[2] Arcos-Álvarez D, Canul-Solís J, García-Herrera R, Sarmiento-Franco L, Piñeiro-Vazquez Á, Casanova-Lugo F, Chay-Canul A (2020). Udder measurements and their relationship with milk yield in Pelibuey ewes. Animals.

[3] Pourlis A (2020). Ovine mammary morphology and associations with milk production, milkability and animal selection. Small Rumin. Res..

[4] Ayadi M, Such X, Ezzehizi N, Zouari M, Najar T, M’Rad MB, Casals R (2011). Relationship between mammary morphology traits and milk yield of Sicilo-Sarde dairy sheep in Tunisia. Small Rumin. Res..

[5] Iñiguez L, Hilali M, Thomas DL, Jesry G (2009). Udder measurements and milk production in two Awassi sheep genotypes and their crosses. J. Dairy Sci..

[6] Rovai M, Caja G, Such X (2008). Evaluation of udder cisterns and effects on milk yield of dairy ewes. J. Dairy Sci..

[7] McKusick, B. C., Marnet, P. G., Berger, Y. M. & Thomas, D. L. Preliminary results: Effects of udder morphology on commercial milk production of East Friesian crossbreed ewes. *Proceedings of the 5th Great Lakes Dairy Sheep Symposium*. November 4–6, 1999, Brattleboro, Vermont, USA (1999).

[8] Van der Linden DS, Lopez-Villalobos N, Kenyon PR, Thorstensen E, Jenkinson CMC, Peterson SW, Blair HT (2010). Comparison of four techniques to estimate milk production in singleton-rearing non-dairy ewes. Small Rumin. Res..

[9] Emediato RMS, Siqueira ERD, Stradiotto MM, Maestá SA, Fernandes S (2008). Relationship between udder measurements and milk yield in Bergamasca ewes in Brazil. Small Rumin. Res..

[10] Espinosa-Mendoza RI, Arcos-Álvarez DN, Garcia-Herrera RA, Antonio-Molina G, Vicente-Pérez R, Macias-Cruz U, Chay-Canul AJ (2020). Predicting milk yield in Pelibuey ewes from the udder volume measurement with a simple method. J. Dairy Res..

[11] Bakoev S, Getmantseva L, Kolosova M, Kostyunina O, Chartier DR, Tatarinova TV (2020). PigLeg: Prediction of swine phenotype using machine learning. PeerJ.

[12] Madhiarasan M, Deepa SN (2016). A novel criterion to select hidden neuron numbers in improved back propagation networks for wind speed forecasting. Appl. Intel..

[13] Li MM, Sengupta S, Hanigan MD (2019). Using artificial neural networks to predict pH, ammonia, and volatile fatty acid concentrations in the rumen. J. Dairy Sci..

[14] Cravener TL, Roush WB (2001). Prediction of amino acid profiles in feed ingredients: Genetic algorithm calibration of artificial neural networks. Anim. Feed Sci. Technol..

[15] Grzesiak W, Lacroix R, Wójcik J, Blaszczyk P (2003). A comparison of neural network and multiple regression predictions for 305-day lactation yield using partial lactation records. Can. J. Anim. Sci..

[16] Usman SM, Singh NP, Dutt T, Tiwari R, Kumar A (2020). Comparative study of artificial neural network algorithms performance for prediction of FL305DMY in crossbred cattle. J. Entomol. Zool. Stud..

[17] Montout, A. X., Bhamber, R. S., Lange, D. S., Ndlovu, D. Z., Morgan, E. R., Ioannou, C. C. & Dowsey, A. W. Accurate and interpretable prediction of poor health in small ruminants with accelerometers and machine learning. *bioRxiv.*10.1101/2020.08.03.234203 (2020).

[18] Ehret A, Hochstuhl D, Gianola D, Thaller G (2015). Application of neural networks with back-propagation to genome-enabled prediction of complex traits in Holstein-Friesian and German Fleckvieh cattle. Genet. Sel. Evol..

[19] Hernández-Ramos PA, Vivar-Quintana AM, Revilla I (2019). Estimation of somatic cell count levels of hard cheeses using physicochemical composition and artificial neural networks. J. Dairy Sci..

[20] Murata N, Yoshizawa S, Amari SI (1994). Network information criterion-determining the number of hidden units for an artificial neural network model. IEEE Trans. Neural Netw..

[21] Bhosale MD, Singh TP (2015). Comparative study of feed-forward neuro-computing with multiple linear regression model for milk yield prediction in dairy cattle. Curr. Sci..

[22] Liseune A, Salamone M, Van den Poel D, Van Ranst B, Hostens M (2021). Predicting the milk yield curve of dairy cows in the subsequent lactation period using deep learning. Comput. Electron. Agric..

[23] Ince D, Sofu A (2013). Estimation of lactation milk yield of Awassi sheep with artificial neural network modeling. Small Rumin. Res..

[24] Fernández C (2007). Weekly milk prediction on dairy goats using neural networks. Neural. Comput. Appl..

[25] Kaygisiz F, Sezgin FH (2017). Forecasting goat milk production in Turkey using Artificial Neural Networks and Box-Jenkins models. Anim. Rev..

[26] Singh NP, Usman SM, Maurya V, Dutt T, Bhatt N, Kumar A (2020). Comparative analysis of artificial neural network algorithms for prediction of FL305DMY in Murrah Buffalo. Int. J. Livest. Res..

[27] Ankinakatte S, Norberg E, Løvendahl P, Edwards D, Højsgaard S (2013). Predicting mastitis in dairy cows using neural networks and generalized additive models: A comparison. Comput. Electron. Agric..

[28] Norouzian MA, Bayatani H, Alavijeh MV (2021). Comparison of artificial neural networks and multiple linear regression for prediction of dairy cow locomotion score. Vet. Res. Forum.

[29] Khorshidi-Jalali M, Mohammadabadi M, Koshkooieh AE, Barazandeh A, Babenko O (2019). Comparison of artificial neural network and regression models for prediction of body weight in Raini Cashmere goat. Iran. J. Appl. Anim. Sci..

[30] Akkol S, Akilli A, Cemal I (2017). Comparison of artificial neural network and multiple linear regression for prediction of live weight in hair goats. Yyu J. Agric. Sci.

[31] Adebayo RA, Moyo M, Gueguim-Kana EB, Nsahlai IV (2020). The use of artificial neural networks for modelling rumen fill. Can. J. Anim. Sci..

[32] Ekiz B, Baygul O, Yalcintan H, Ozcan M (2020). Comparison of the decision tree, artificial neural network and multiple regression methods for prediction of carcass tissues composition of goat kids. Meat Sci..

[33] Dallago GM, de Figueiredo DM, de Resende Andrade PC, dos Santos RA, Lacroix R, Santschi DE, Lefebvre DM (2019). Predicting first test day milk yield of dairy heifers. Comput. Electron. Agric..

[34] Nobari K, Baneh H, Esmaeilkhanian S, Yussefi K, Samiei R (2019). Comparison of linear model and artificial neural network to prediction of milk yield using first recorded parity. J. Rumin. Res..

[35] Garson GD (1998). Neural Networks: An Introductory Guide for Social Scientists.

[36] Zeng X, Yeung DS (2006). Hidden neuron pruning of multilayer perceptrons using a quantified sensitivity measure. Neurocomputing.

[37] Fritsch S., Guenther F. & Wright M. N. (2019). neuralnet: Training of Neural Networks. R package version 1.44.2. https://CRAN.R-project.org/package=neuralnet

[38] Akaike H (1974). A new look at the statistical model identification. EEE Trans. Autom. Control.

[39] Bhat HS, Kumar N (2010). On the derivation of the bayesian information criterion. School Nat. Sci. Univ. California.

